# Exploring new battery knowledge by advanced characterizing technologies

**DOI:** 10.1002/EXP.20210130

**Published:** 2021-12-28

**Authors:** Liguang Wang, Tiefeng Liu, Tianpin Wu, Jun Lu

**Affiliations:** ^1^ Advanced Photon Sources X‐Ray Science Division Argonne National Laboratory Lemont Illinois USA; ^2^ Department of Chemistry and Biochemistry University of Windsor Windsor Ontario Canada; ^3^ College of Materials Science and Engineering Zhejiang University of Technology Hangzhou P. R. China; ^4^ Chemical Sciences and Engineering Division Argonne National Laboratory Lemont Illinois USA

**Keywords:** battery materials, solid‐electrolyte interphase, transmission X‐ray microscopy and cryo‐transmission electron

## Abstract

Exploration of science and technologies represents human's thirst for new knowledge and new life. Presently, we are in a stage of transferring the use of fossil fuels to renewable energy, which urgently calls for new energy materials and techniques beyond the boundary of human knowledge. On the way of scrutinizing these materials and surmounting the bottleneck of their performances, characterizing technologies are of critical importance in enabling the revealing of materials regarding their structural and chemical information, eventually establishing the correlations between microstructures and properties at the multiscale levels. Regrettably, traditional characterizations are hard to simultaneously probe electrochemistry with these chemical and physical structural evolutions, especially under operando conditions, or offer high‐resolution images of materials sensitive to electron‐beam irradiation. To this end, various advanced characterizing and diagnosing technologies recently developed, such as transmission X‐ray microscopy and cryo‐transmission electron microscopy, have demonstrated their benefits in understanding the energy storage behaviors of high‐performance energy materials (such as layered transition oxide cathode and Li metal anode). Benefited from new knowledge, the progress of high‐capacity electroactive materials is significantly accelerated. Here, we timely review the breakthroughs in emerging techniques and discuss how they guide the design of future battery materials to achieve the ultimate carbon neutrality.

## Graphical abstract

Advanced characterizing technologies are of critical importance in enabling the revealing of materials regarding their structural and chemical information, eventually establishing the correlations between microstructures and properties at the multiscale levels.

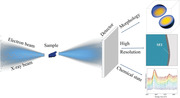

## 1 INTRODUCTION

Energy is the foundation and driving force for the progress of human civilization through satisfying basic social needs and spurring economic growth.^[^ ^1^ ^]^ In the 20th century, fossil fuels are preferentially utilized to propel the industrial revolution but regretfully entail the excessive emission of greenhouse gas and so‐called global warming. Entering the 21st century, advances in technologies enable access to renewable energy sources such as solar, wind, and tide energy.^[^ ^2^ ^]^ Today, the realm of energy conversion and storage has become a hot spot for governmental funding and corporate investment. Exploring the suitable ways for renewable energy capturing and storage on large scale has been the mandate of the scientists and engineers to cope with the drastic energy demand and pressing expansion of modern industries.

Rechargeable battery technology is rooted in a series of electrochemical principles regarding storing electrical energy via the chemical reactions between the anode and cathode, and releasing the electrical energy through the reverse process when needed.^[^ ^3^ ^]^ Currently, the applications of rechargeable batteries are ubiquitous, to name a few, mobile electronics, electric vehicles, and smart grids. Most typically, the rechargeable batteries based on lithium (Li)‐ion intercalation chemistries have achieved a great success in commercialization relying on their advantages in specific energy, rate capability, cycle life, and abuse tolerance.^[^ ^4^ ^]^ Furthermore, research into Li‐ion batteries (LIBs) has become rather attractive and climbed sharply in popularity, resulting in the birth of new Li‐storage materials and the more fundamental insight into electrodes and electrolyte.^[^ ^5^ ^]^ In this context, understanding both the changes upon the structure and chemistry of electroactive materials during energy storage is of critical importance, which urgently calls for advanced techniques for in‐depth characterizing and diagnosing of electroactive materials.

In this perspective, we pay attention to the progress of advanced characterizations in deepening the fundamental understanding and fostering further development of high‐performance batteries. Specific focus is placed on transmission X‐ray microscopy (TXM) and cryo‐transmission electron microscopy (cryo‐TEM), both of which are increasingly accessible to the battery scientists. As of now, these two techniques have become mature enough to help us reveal key structural and chemical information of battery materials. Thus, we first discuss the specific capability of synchrotron‐based X‐ray imaging techniques in LIBs. Subsequently, the significant advances in the exploration of atomic structure and chemical constituents of the Li metal anode are highlighted. We believe that these fundamental insights into battery materials enabled by emerging characterization techniques are of great significance for boosting their electrochemical performances.

## 2 MULTISCALE X‐RAY IMAGING MECHANOCHEMISTRY CHANGES OF BATTERY MATERIALS

**FIGURE 1:**
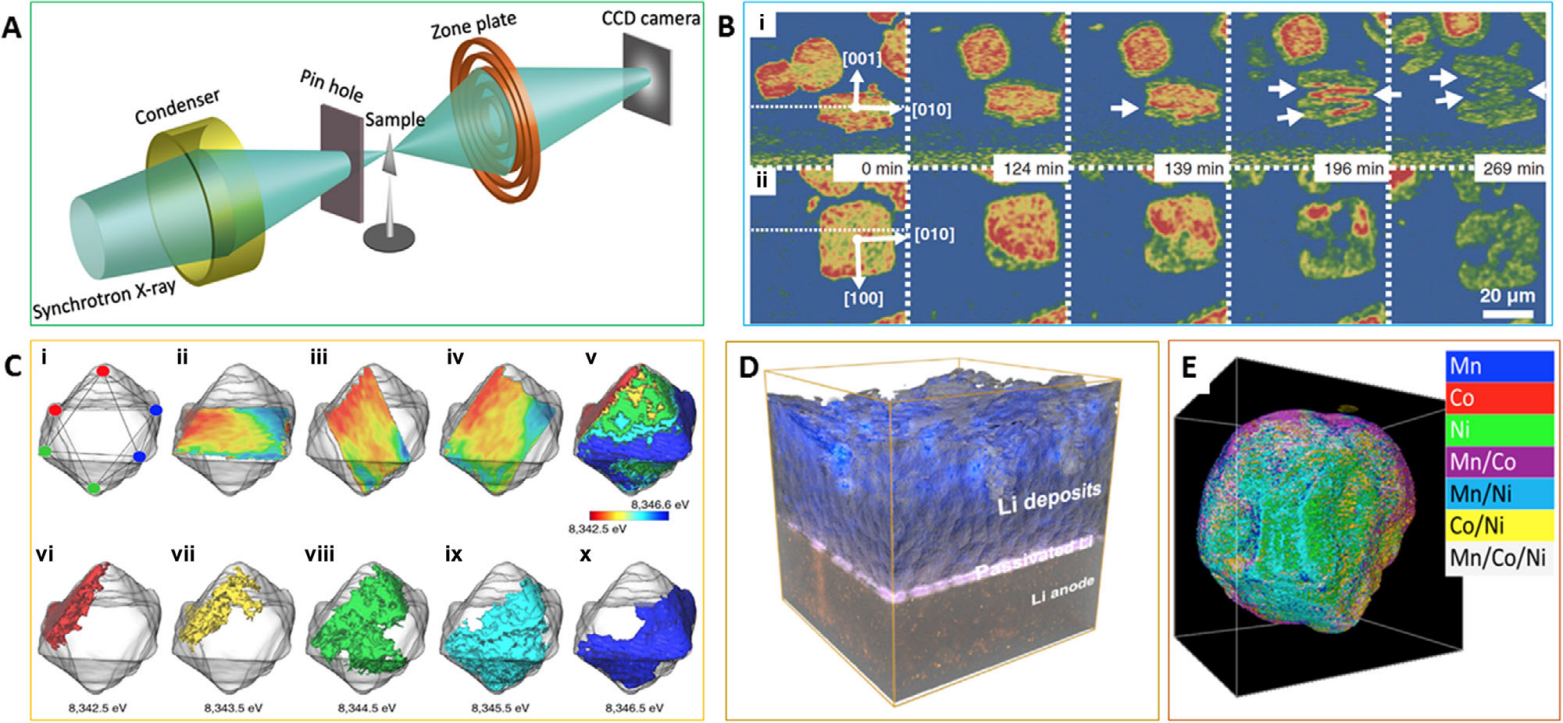
Real‐space X‐ray imaging techniques. (A) Schematic illustration of the TXM experimental setup. Reproduced with permission.^[^ ^13^ ^]^ Copyright 2017, Elsevier. (B) Coronal and transverse cross sections through a particle during electrochemical reduction. Horizontal dotted white lines in the cross sections at 0 min indicate cutting planes. Reproduced with permission.^[^ ^10^ ^]^ Copyright 2013, AAAS. (C) The shape of the particle is presented as the transparent grey surface with the internal oxidation state heterogeneity illustrated using the diagonal slices surfaces of the 3D Ni oxidation state map. Reproduced with permission.^[^ ^15^ ^]^ Copyright 2017, Nature Publish Group. (D) 3D volume rendering the image including Li deposits and a Li anode. Reproduced with permission.^[^ ^12^ ^]^ Copyright 2021, American Chemical Society. (E) 3D Elemental sensitivity nano‐tomography reveals the transition metal segregation within a secondary particle. Reproduced with permission.^[^ ^16^ ^]^ Copyright 2016, Nature Publish Group

Since the late 19th century, X‐rays have been applied for imaging at the first discovery. Attributing to the excellent penetration capability, X‐ray microscopy can directly and non‐destructively visualize the mechanochemistry changes within battery materials under operando conditions. Various setup and principles of the imaging systems bring two categories of X‐ray microscopic techniques that are real‐space and reciprocal space imaging methods.^[^ ^6^ ^]^ Here, the real‐space imaging techniques, mainly the TXM technique (Figure 1A), are selected to probe the electrochemistry in battery materials. The images obtained by the real space X‐ray imaging techniques provide direct observation, which is straightforward for analysis. However, the spatial resolution of these methods is usually determined by the optics in the system. For soft X‐rays, the spatial resolution may reach the nanometer level, while for hard X‐rays, the resolution is slightly worse.^[^ ^7^ ^]^ One common synchrotron‐based X‐ray microscope is very similar to the traditional visible light microscope, by which a full‐field image can be achieved at the focused plane through a two‐dimensional area detector behind the sample. This imaging method is commonly referred to as full‐field TXM.^[^ ^8^ ^]^ Another common real‐space imaging technique is built up point‐by‐point by scanning the sample across the interest area, which is generally called the scanning imaging method.^[^ ^9^ ^]^ Both methods can investigate the electronic and morphologic changes with respect to different data processing principles. These non‐destructive imaging techniques can track the mechanochemistry changes from multiscale (from nanometers to electrode level) upon operando conditions and may lead to interesting findings that are previously hard to discover by conventional approaches.^[^ ^10^ ^]^ We will discuss the specific capability of synchrotron‐based real‐space imaging techniques in secondary batteries in the following section.

The Fresnel‐zone‐plate‐based full‐field TXM techniques are believed as a powerful tool in terms of non‐destructive projections acquisition speed with a spatial resolution of tens of nanometers, especially when applied to the advanced high brightness synchrotron light source.^[^ ^11^ ^]^ One typical example is the *in operando* observation of electrochemical and mechanical degradation in the LIBs. An electrode‐level investigation on the individual particles was conducted on the model material of tin oxide to reveal the origins and evolution of electrochemical and mechanical degradation (Figure 1B).^[^ ^10^ ^]^ Compared to penetration capability limited traditional imaging techniques, such as transmission/scanning electron microscopy, atomic force microscopy, etc., X‐ray tomography achieved by using the full‐field TXM technique provides a three‐dimensional (3D) chemical composition and morphology on single‐particle throughout the whole electrode, raveling the lithiation mechanism, crack initiation and growth behavior during the following lithiation process. It is worth noting that this direct record image is highly related to the X‐ray attenuation coefficient, leading to high sensitivity to high atomic number *Z* but low *Z*. The utilization of phase‐contrast imaging technique that is determined by the variations in intensity can offset this disadvantage. The tracking of Li plating/stripping behavior in battery systems, especially upon operando conditions, has always been a challenge given the high activity and sensitivity of the Li element with a low atomic number. To this end, the phase‐contrast X‐ray imaging technique holds promise to provide an operando observation of the Li electrochemical behavior in practical battery systems. This is because the atomic number *Z* sensitivity of the phase‐contrast imaging method can distinguish the light Li element from other battery materials. For instance, the formation of porous structure and the corresponding progression revealed can significantly determine the subsequent Li plating and lead to the topologic inhomogeneity of Li metal anode (Figure 1D).^[^ ^12^ ^]^

Further coupling with X‐ray absorption near edge structure (XANES) by using a continuous tunable synchrotron X‐ray source, the X‐ray spectroscopic imaging can offer chemical phase mapping that demonstrates the spatial resolved electronic structure of the mapping elements. This X‐ray spectroscopic imaging method has demonstrated its advantage in the field of battery research, where the morphological and chemical changes occur simultaneously. By leveraging the capacity of XANES analysis, TXM can give the chemical oxidization state mappings with high chemical and elemental sensitivity at a high resolution of tens of nanometers, which is critical for understanding the reaction mechanism in battery materials under *in operando* electrochemical conditions. By using the *in operando* two‐dimensional (2D) TXM‐XANES mappings, we revealed the core‐shell reaction mechanism in the layered oxides that experience small volume changes during the whole electrochemical cycle.^[^ ^13^ ^]^ For the electrode materials with large volume changes, another digging reaction mechanism was also demonstrated on the nickel sulfide anode material by the combination of the morphological and chemical evolution on the single‐particle.^[^ ^14^ ^]^

It is worth emphasizing that this spectroscopic imaging approach can also resolve the 3D elemental/chemical distribution when coupled with tomography techniques. The utilization of 3D spectroscopic imaging method can provide detailed structural information inside the active materials, such as the effect of crystal orientation and composition gradient design. For example, by combining the 2D/3D chemical phase mappings, Kuppan et al., revealed a three‐phase transformation mechanism that is conflicting with the well‐known particle‐by‐particle or shrinking‐core reaction mechanism during the (de)lithiation process in the single‐crystal spinel oxides cathode (Figure 1C).^[^ ^15^ ^]^ The transition metal segregation induced electrochemical degradation in layered structure oxides was disclosed by the energy‐resolved full‐field X‐ray nanotomography which provided the distribution of all the transition metals (Ni, Co, and Mn) within the particles (Figure 1E).^[^ ^16^ ^]^ These in‐depth understandings revealed by the advanced 3D spectroscopic imaging technique demonstrate the strong capability of the combination of 3D nano‐tomography and spectroscopy to probe the electrochemical behaviors in battery materials.

## 3 ATOMIC IDENTIFICATION OF SENSITIVE INTERFACES IN BATTERY MATERIALS

**FIGURE 2:**
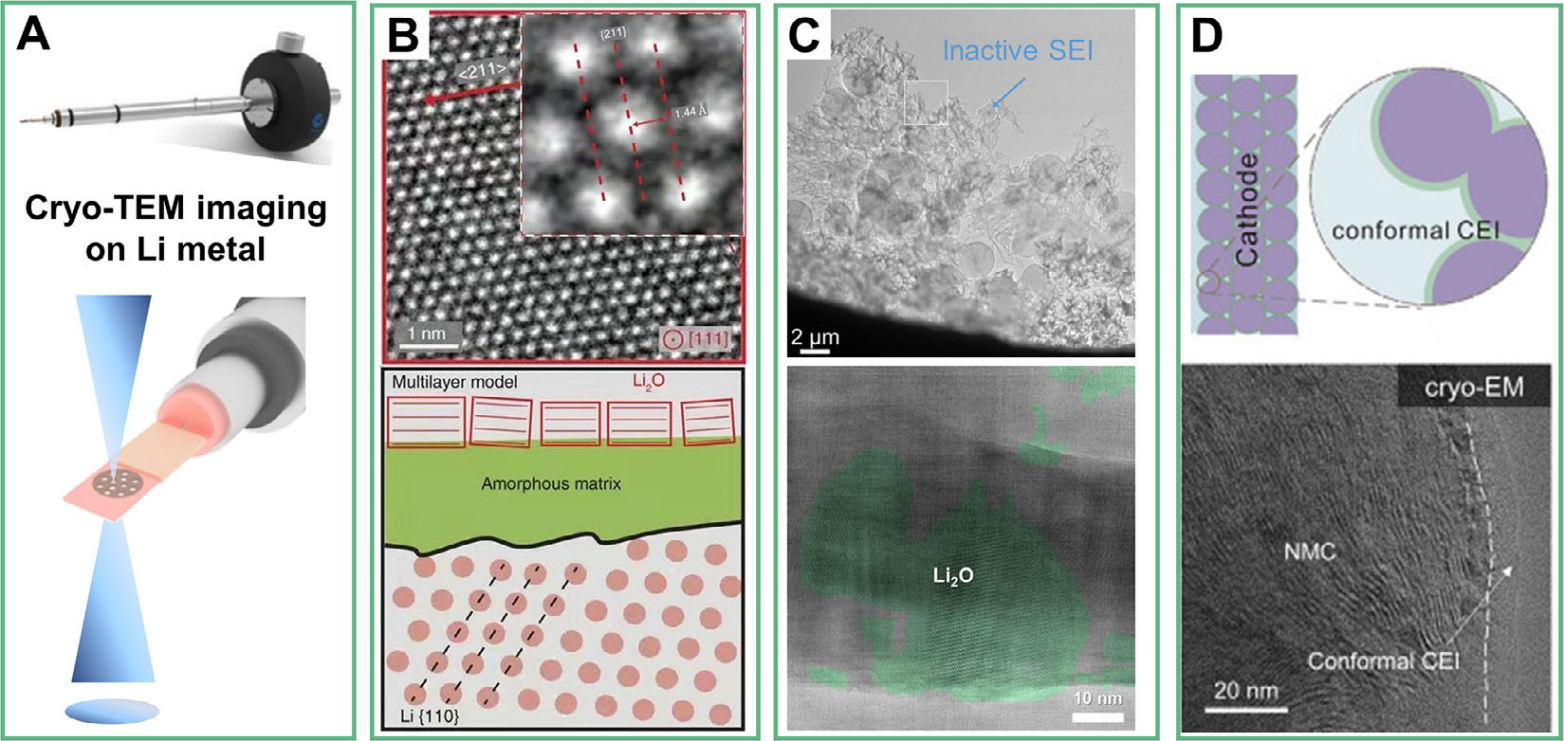
Cryo‐TEM techniques. (A) the schematic illustration of Cryo‐TEM, (B) Cryo‐TEM images of Li dendrites at atomic resolution along the [111] zone axis. Reproduced with permission.^[^ ^22^ ^]^ Copyright 2017, AAAS. (C) Microstructures and components of different SEIs formed on Li metal surface in ether electrolytes. Reproduced with permission.^[^ ^21^ ^]^ Copyright 2021, Nature Publish Group. (D) The schematic diagram for the conformal CEI formed on the cathode surface and corresponding Cryo‐TEM images. Reproduced with permission.^[^ ^28^ ^]^ Copyright 2021, Elsevier

Despite the 3D imaging technique being impressive, the battery materials need to bear high‐energy electron beam irradiation from TXM. Notably, there are also substantial metastable constituents within the LIBs.^[^ ^17^ ^]^ Typically, solid electrolyte interphase (SEI) was earlier proposed by Peled et al. in 1979.^[^ ^18^ ^]^ Given the natural feature of the SEI itself being chemically reactive and sensitive to electron‐beam irradiation, its atomic structure and distribution are difficult to observe by conventional characterizing technologies, such as TEM and X‐ray diffraction. The real SEI structure, especially for organic phase, is rapidly destroyed by the thermal effect stemming from high‐energy electron beam irradiation.^[^ ^19^ ^]^ Therefore, in most cases, the X‐ray photoelectron spectroscopy (XPS) is a commonly used tool to study chemical components. However, probing an SEI structure from XPS alone is still challenging as it fails to reveal in‐plane heterogeneity at the submicron scale.^[^ ^20^ ^]^ The local chemical and structural information in SEI layer remain so far unknown, making the SEI layer very mysterious and becoming the least understood aspect of the battery realm. Currently, evidence is mounting that understanding the formation and evolution of the SEI layer is indispensable because of its unique role in regulating the electrolyte corrosion rate, the kinetic parameters of Li diffusion, and interfacial stability.^[^ ^21^ ^]^ Until recently, the emerging Cryo‐TEM technique was proposed to characterize the detailed atomic structure of Li metal and its SEI layer (Figure 2A,B).^[^ ^19^, ^22^ ^]^

So far, utilizing the Cryo‐TEM technique has become a cutting‐edge study involving the atomic characterization for metallic Li anode.^[^ ^19^ ^]^ First of all, all the operations for the targeted samples should be handled in the inert atmosphere and the sample is sealed in the holder tip. Subsequently, the cryo‐TEM by using liquid nitrogen cooling can retain the sample temperature below −170 °C and in an effort to reduce the thermal damage to the sample during high‐resolution imaging process. When their original state and structure are effectively retained without electron beam damage, further observation at the micro/nanoscale, or even at the atomic scale is possibly implemented. Correspondingly, the resultant discoveries could reshape the knowledge regarding the formation and evolution of the extrinsic interface. For example, the discrepancy between LiF‐dominated SEI on the surface of graphite anode and Li_2_O‐rich SEI derived from Li metal anode is intentionally described. For the graphite anode, LiF is the well‐recognized main composition of the SEI layer, which is the result of chemical and electrochemical reactions on anode surface at the initial discharging process.^[^ ^23^ ^]^ However, such an understanding of the SEI layer does not apply to the Li metal anode. Considering the highly reductive nature of Li metal, very recent studies assisted by cryo‐TEM have provided convincing evidence that Li_2_O instead of LiF is the dominant component in the SEI (Figure 2C).^[^ ^20^, ^22^, ^24^ ^]^ Furthermore, the Li_2_O content in the SEI layer also determines its detrimental role in producing electrically isolated dead Li metal which is principally responsible for the performance decay commonly observed in Li metal batteries.^[^ ^21^ ^]^ Therefore, such a discrepancy renders novel strategies to deal with Li_2_O‐based dead SEI. For example, a Li restoration method based on iodine redox chemistry is proposed to effectively rejuvenate electrochemically inactive Li species in both the redundant SEI and dead electrically isolated Li metal debris.^[^ ^21^ ^]^

Besides, the Cryo‐TEM technique is also applicable to probe the underlying mechanism of why certain electrolyte formula allows the electroactive materials to have successful performances.^[^ ^25^ ^]^ Typically, silicon (Si)‐based materials to be used for stable Li storage require the cooperation of using fluoroethylene carbonate (FEC) as electrolyte additive.^[^ ^26^ ^]^ An amorphous SEI was formed during lithiation, consisting of organic poly(VC) and inorganic Li*_x_* SiO*_y_* layers. While in the subsequent delithiation process, a conformal surface layer remains, which is attributed to the stable poly(VC) components against the oxidation and therefore, contributing to the excellent stability of the Si anode in the FEC‐based electrolyte system.^[^ ^27^ ^]^ This work bridges the gap between microscale and atomic‐scale characterizations of battery electrodes, revealing the nanoscale and mesoscale heterogeneity of extrinsic interphases. Meanwhile, the Cryo‐TEM technique is also a powerful tool for direct imaging and characterization of cathode electrolyte interphase (CEI) layer preserved in native state at atomic scale since the critical role of CEI on cathode surface in preventing parasitic side reactions is increasingly emphasized. The related information will lay the fundamental for understanding the electrode evolution and battery‐failure mechanisms. New insights in CEI would help make the rational design of electrodes and electrolytes for emerging battery chemistry possible. For instance, a facile strategy based on a brief external electrical shorting design between anodes and cathodes and SEI evolution is proposed to achieve the conformal CEI layer on cathode surface.^[^ ^28^ ^]^ The established CEI help boosts the Coulombic efficiency and capacity retention of the cathode during the repeated cycling (Figure 2D).

Although Cryo‐TEM provides a possibility in observing Li metal and its surface by to a great extent eliminating thermal damage from electron beam to SEI layer, the long‐time irradiation will still lead to image floating and deformation, especially for organic phase in SEI layer.^[^ ^19^ ^]^ In a future trend, low‐dose imaging technology at cryogenic temperatures further retains its original state and structure from electron beam damage, and also can be imaged at the micro/nanoscale, or even at the atomic scale. In this context, the more accurate information is possibly revealed due to the iterative upgrading of characterizing technology. The fundamental insight into interfacial chemistries in battery materials is imperative for inhibiting uncontrollable dendrite growth and inactive Li formation in future research.

## 4 SUMMARY

In summary, the state‐of‐the‐art characterizations including cryo‐TEM and TXM highlighted in this perspective can probe the evolutions of physical and chemical properties at multi‐length scales from atomic to the electrode level, especially under the operando conditions. Coupling various techniques determine a powerful tool for comprehensively understanding the structural changes in battery materials. Further development of electron and/or X‐ray sources such as much brighter synchrotron radiation could shorten the time of data collection, which makes the real‐time observation and high spatial resolution possible. Other advanced spectroscopic, imaging, scattering, etc., techniques can also provide critical complementary information that will make significant contributions to develop next‐generation battery systems.

## CONFLICT OF INTERESTS

The authors declare no competing financial interests.
